# Association between the body roundness index and all-cause mortality in patients with metabolic dysfunction- associated steatotic liver disease

**DOI:** 10.3389/fpubh.2026.1765588

**Published:** 2026-02-20

**Authors:** Zechao He, Fei Tian, Jianguo Jia, Hong Ji, Yunpeng Li, Xinyu Ge, Shuanghao Zhou, Jiahui Zou, Ze Wang, Yurui Du, Xueying Ma, Xiangming Ma

**Affiliations:** 1 Department of Hepatobiliary Surgery, Kailuan General Hospital, North China University of Science and Technology, Tangshan, China; 2 Department of Radiation Oncology, North China University of Science and Technology Affiliated Hospital, Tangshan, China

**Keywords:** all-cause mortality, body roundness index (BRI), cohort study, epidemiology, metabolic dysfunction-associated steatotic liver disease (MASLD)

## Abstract

**Background:**

The body roundness index (BRI) is a novel anthropometric measure derived from waist circumference and height that reflects abdominal adiposity. Previous studies have demonstrated that BRI has predictive value for all-cause mortality in the general population and in individuals with metabolic dysfunction–associated steatotic liver disease (MASLD) in the United States. However, the association between BRI and all-cause mortality in patients with MASLD from northern Chinese populations remains unclear.

**Methods:**

In this population-based prospective cohort study, we analyzed 28,898 MASLD patients (mean age 52.3 ± 12.2 years) from the Kailuan Study, an ongoing longitudinal investigation of Chinese industrial workers. The primary outcome was all-cause mortality. The Cox proportional hazards regression model was utilized to assess the association between BRI and the risk of all-cause mortality in the MASLD population by calculating hazard ratios (HR) with 95% confidence intervals (CI).

**Results:**

During a median follow-up of 13.62 years (interquartile range 12.85–15.16), 3,895 deaths were occurred. After adjustment for confounders, each standard deviation increase in BRI was associated with a 13% increased risk of all-cause mortality (HR = 1.13, 95% CI: 1.07–1.19, *P* < 0.001). Multivariable Cox regression analysis revealed that compared with subjects in the lowest BRI quartile (Q1), those in the third (Q3) and fourth (Q4) quartiles had hazard ratios for all-cause mortality of 1.14 (95% CI: 1.02–1.28) and 1.21 (95% CI: 1.06–1.37) (*P* for trend < 0.001), respectively.

**Conclusion:**

BRI demonstrated a positive association with all-cause mortality in the MASLD population.

## 1 Introduction

Metabolic dysfunction-associated steatotic liver disease (MASLD) (1) is a disease entity jointly proposed and updated by three major international hepatology societies. This condition was previously referred to as non-alcoholic fatty liver disease (NAFLD) and metabolic-associated fatty liver disease (MAFLD) (2). With advances in research, NAFLD has increasingly been recognized as not merely a liver-specific disorder but a multisystem disease (3) characterized by insulin resistance, multiple metabolic abnormalities, and extrahepatic complications (4, 5). At the same time, the terminology MAFLD has been considered to carry potential social stigma to some extent (1). MASLD has emerged as the most prevalent chronic liver condition globally, affecting approximately 30% of the population and driving substantial healthcare utilization related to end-stage liver disease and hepatic complications (6–9). Distinct from NAFLD diagnostic criteria, MASLD emphasizes cardiometabolic risk stratification through standardized evaluation of body mass index, glycemic status, blood pressure, and lipid profiles (1). Prior studies have consistently demonstrated a significant association between MASLD and an elevated risk of all-cause mortality (10–12). Consequently, optimizing the management of MASLD patients to mitigate all-cause mortality is critically needed.

Traditional obesity assessment primarily relies on body mass index. While extensive evidence confirms that BMI-defined obesity confers significantly elevated all-cause mortality risks compared to normal BMI ranges (13), growing recognition of body composition has shifted research focus toward visceral adiposity-mortality associations (14, 15). BMI's fundamental limitation lies in its inability to differentiate fat distribution patterns—individuals with identical BMIs may exhibit markedly divergent visceral adipose tissue accumulation and muscle mass proportions (16). To address this gap, the Body Roundness Index (BRI), developed by Thomas et al. (17), provides a geometrically derived metric integrating waist circumference and height to better quantify central adiposity patterns.

Current evidence positions the Body Roundness Index (BRI) as a superior anthropometric predictor of clinical endpoints compared to conventional indices, demonstrating enhanced risk stratification capacity for cardiometabolic diseases (18, 19), kidney disease (20), and malignancy (21). Furthermore, previous studies in the general US. population have reported a U-shaped association between the body roundness index (BRI) and all-cause mortality (22), and this relationship has also been examined in U.S. populations with metabolic-associated fatty liver disease (23). To date, the association between Body Roundness Index (BRI) and all-cause mortality in Chinese adults with metabolic dysfunction–associated steatotic liver disease (MASLD) has been rarely investigated. In addition, evidence regarding the potential role of BRI in risk stratification among this population remains limited. Therefore, using data from the Kailuan cohort in northern China, we evaluated the association between BRI and all-cause mortality in Chinese adults with MASLD.

## 2 Methods

### 2.1 Study design and participants

**Figure 1:**
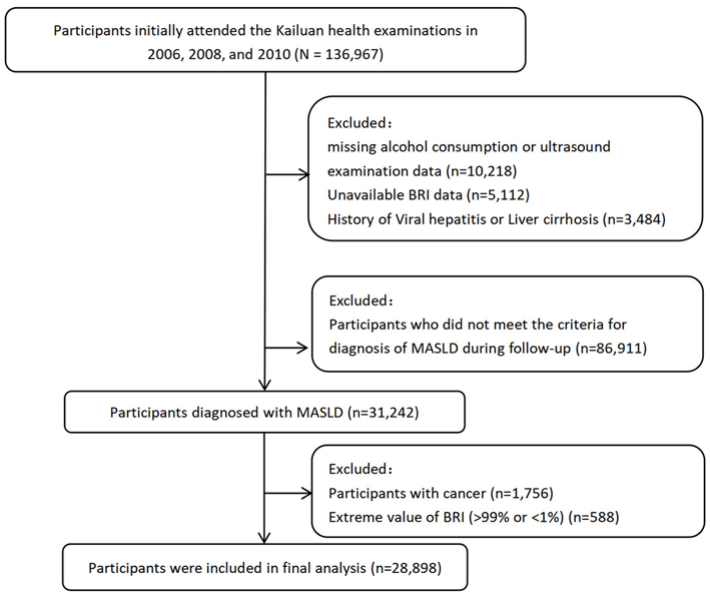
Workflow of participant recruitment and screening. BRI, body roundness index; MASLD, metabolic dysfunction-associated steatotic liver disease.

This investigation utilized data from the Kailuan Study, an ongoing population-based longitudinal investigation initiated in 2006 with biennial follow-up assessments capturing updated demographic, laboratory, imaging, and lifestyle parameters as previously detailed (24). From the baseline population of 136,967 participants who underwent initial health examinations between 2006–2007, 2008–2009, and 2010–2011, we excluded individuals with: 1) missing baseline BRI data; 2) missing alcohol consumption records or ultrasound examination data; 3) History of viral hepatitis or liver cirrhosis; 4) failure to meet MASLD diagnostic criteria; 5) malignancy; and 6) extreme BRI values (>99% or < 1%). After exclusions, 28,898 eligible MASLD patients were included in the final cohort (Figure 1). This study was conducted in accordance with the Declaration of Helsinki and received ethical approval from the Institutional Review Board of Kailuan General Hospital (Approval No: 2006-05). All participants provided written informed consent.

### 2.2 Anthropometric measurements

Trained personnel obtained height, weight, and waist circumference (WC) using standard protocols. Height and WC were measured to the nearest 0.1 cm, with weight recorded to 0.1 kg using calibrated platform scales. Participants wore light indoor clothing without shoes during measurements. WC was assessed at the umbilicus level with non-stretchable tape at the end of normal expiration. Body Roundness Index (BRI) was calculated as follows (17).$BRI=364.2-365.5\times \sqrt{1-\frac{{\left(WC/2\pi \right)}^{2}}{{\left(0.5\times height\right)}^{2}}}$

### 2.3 Definition of MASLD

The diagnosis of MASLD followed a two-stage protocol. First, all participants underwent abdominal ultrasonography for hepatic steatosis assessment. The details of the ultrasound procedure for diagnosing hepatic steatosis are as described previously (24). Hepatic steatosis is diagnosed by ultrasound in the presence of at least two of the following conditions: (1) diffuse increased echogenicity of the liver relative to the kidney, (2) attenuation of the ultrasound beam, and (3) poor visualization of intrahepatic structures, and classified into three categories: mild (diffuse increase in fine echogenicity in the liver parenchyma), moderate (diffuse increase in fine echogenicity with impaired visualization of intrahepatic vascular borders and diaphragm), and severe (diffuse increase in fine echogenicity with no visibility of intrahepatic vascular borders and diaphragm). In the Kailuan Study, standardized abdominal ultrasounds were performed by certified radiologists using high-resolution B-mode ultrasound systems (ACUSON X300; Siemens Healthineers, Germany) equipped with 3.5 MHz convex-array transducers.

Participants diagnosed with hepatic steatosis who denied excessive alcohol consumption (ethanol intake ≥210 g/week for men or ≥140 g/week for women) subsequently underwent metabolic evaluation. A diagnosis of MASLD requires the presence of at least one of five cardiometabolic risk factors (CMRFs) (1): MASLD was defined as the presence of SLD and one or more of the following cardiometabolic risk factors: (1) body mass index ≥23kg/m2 or waist circumference ≥90cm (for males) or≥80cm (for females); (2) fasting glucose ≥100mg/dl or type 2 diabetes or glucose-lowering drug use; (3) BP≥130/85 mm Hg or BP-lowering drug use; (4) triglyceride ≥150mg/dl or lipid-lowering drug use; (5) high-density lipoprotein cholesterol < 40mg/dl (for males) or < 50mg/dl (for females) or lipid-lowering drug use. Participants demonstrating hepatic steatosis with ≥1 cardiometabolic risk factor (CMRF) were classified as MASLD cases after exclusion of secondary causes of steatosis.

### 2.4 Outcomes

The primary outcome was all-cause mortality. The follow-up period was defined as the time from baseline until the occurrence of death or the end of the follow-up period (December 31, 2021). All-cause mortality was ascertained annually by medical professionals using death certificates obtained from provincial vital statistics offices.

### 2.5 Covariates assessment

Epidemiological data encompassing anthropometric measurements, lifestyle factors, personal medical history, medication use (including glucose-lowering, antihypertensive, and lipid-modifying agents), and a history of cardiovascular disease were collected through face-to-face interviews using standardized structured questionnaires. Educational attainment was categorized as below high school or high school and above. Smoking status was classified as current or never/former smoker. Seated systolic blood pressure (SBP) was measured twice at 5-min intervals on the left upper arm using calibrated mercury sphygmomanometers after at least 5 mins of rest, with the average value used for analysis. Anthropometric measurements included height, weight, waist circumference, and hip circumference. Fasting blood samples were collected after overnight fasting and analyzed at the central laboratory of Kailuan General Hospital using standardized protocols and automated analyzers (Hitachi 747). Laboratory measurements included neutrophil count (NEUT), high-density lipoprotein cholesterol (HDL-C), high-sensitivity C-reactive protein (Hs-CRP), fasting blood glucose (FBG), uric acid (UA), and creatinine (Cr). Hepatitis B surface antigen (HBsAg) status was assessed using chemiluminescence immunoassays. Covariates were selected *a priori* based on biological plausibility and existing literature as potential confounders of the association between BRI and all-cause mortality. These adjustments aimed to account for baseline cardiometabolic, inflammatory, lifestyle, and socioeconomic factors that may influence both body fat distribution and mortality risk, while minimizing overadjustment by avoiding variables that clearly lie on the causal pathway.

### 2.6 Statistical analysis

Data from standardized physical examinations were entered by trained personnel into an Oracle 10.2g database hosted at Kailuan General Hospital. Analyses were performed using SAS version 9.4. Normally distributed continuous variables are expressed as mean ± standard deviation, with between-group comparisons assessed by one-way ANOVA. Non-normally distributed data are reported as median (25th−75th percentiles) using Kruskal-Wallis H tests. Categorical variables are presented as counts (%) with chi-square tests for group comparisons.

Participants were stratified into quartiles (Q1-Q4) based on BRI values. All-cause mortality rates were calculated per 1,000 person-years. Cumulative all-cause mortality probabilities were estimated using Kaplan-Meier curves with between-quartile differences assessed by log-rank tests. Dose-response relationships between continuous BRI and all-cause mortality risk were modeled using restricted cubic splines (RCS) with 3 knots at the 10th, 50th, and 90th percentiles. Multivariable Cox proportional hazards models estimated hazard ratios (HRs) and 95% confidence intervals (CIs) for the association between BRI and all-cause mortality after sequential adjustment: Model 1 (unadjusted), Model 2 (age and gender adjusted), Model 3 (fully adjusted for clinical/lifestyle covariates). The proportional hazards assumption was formally assessed using Schoenfeld residuals, and no significant violations were detected. Prespecified subgroup analyses were conducted to examine potential effect modification across clinically relevant strata within the MASLD population. A sensitivity analysis was conducted to assess the robustness of the findings. Participants who died within the first 2 years of follow-up were excluded, while participants with cancer at baseline were retained. Missing values for covariates were handled using multiple imputation by chained equations, assuming data were missing at random. All statistical tests were two-sided, and a *P* value < 0.05 was considered statistically significant.

## 3 Results

### 3.1 Comparison of baseline data of groups

This study enrolled 28,898 individuals with metabolic dysfunction-associated steatotic liver disease (MASLD) with a mean age of 52.34 ± 12.20 years (SD), comprising 22,998 male and 5,900 female participants (Figure 1). Participants were stratified into BRI quartiles: Q1 (BRI ≤ 3.64; *n* = 7,202), Q2 (3.64 < BRI ≤ 4.28; *n* = 7,276), Q3 (4.28 < BRI ≤ 5.04; *n* = 7,184), and Q4 (BRI >5.04; *n* = 7,236). Clinically significant between-quartile differences (*P* < 0.05) were observed for age, gender, Hs-CRP, FBG, HDL-C, CR, SBP, UA, Neutrophil, hip circumference, smoking status, cardiovascular disease history, hypertension history, and educational attainment (Table 1).

**Table 1:** Baseline characteristics of the participants according to the quartiles of BRI in MASLD.

**Characteristic**	**Overall**	**Q1 (*n* = 7,202)**	**Q2 (*n* = 7,276)**	**Q3 (*n* = 7,184)**	**Q4 (*n* = 7,236)**	***P* value**
Age (years)	52.34 ± 12.2	49.16 ± 11.4	50.94 ± 11.9	53.16 ± 12.1	56.12 ± 12.3	< 0.001
**Gender [*****N*** **(%)]**			
Male	22,998 (79.6)	5,992 (83.2)	5,964 (82.0)	5,776 (80.4)	5,265 (72.8)	< 0.001
Female	5,900 (20.4)	1,209 (16.8)	1,312 (18.0)	1,408 (19.6)	1,971 (27.2)	
Hs-CRP (mg/L)	1.3 (0.3–3.2)	1.0 (0.4–2.3)	1.2 (0.5–2.8)	1.4 (0.6–3.4)	2.0 (0.9–4.7)	< 0.001
FBG (mmol/L)	5.89 ± 2.02	5.80 ± 1.90	5.85 ± 2.00	5.91 ± 2.00	6.04 ± 2.16	< 0.001
HDL-C (mmol/L)	1.49 ± 0.41	1.53 ± 0.41	1.48 ± 0.39	1.48 ± 0.40	1.48 ± 0.42	0.042
Cr (umol/L)	93.25 ± 32.44	96.81 ± 36.99	93.44 ± 29.82	92.18 ± 32.52	90.58 ± 29.59	< 0.001
SBP (mmHg)	136 ± 20.85	133.77 ± 19.94	135.16 ± 20.53	137.47 ± 21.06	141.32 ± 21.06	< 0.001
UA (umol/L)	306.86 ± 88.66	293.36 ± 83.12	305.35 ± 87.35	313.05 ± 89.56	315.66 ± 92.63	< 0.001
Neutrophil count (109^/L)	4.07 ± 3.38	3.99 ± 2.08	4.09 ± 5.41	4.03 ± 2.21	4.15 ± 2.64	< 0.001
Hip circumference (cm)	101.50 ± 8.56	94.54 ± 6.58	99.41 ± 6.06	103.28 ± 6.53	108.78 ± 7.83	< 0.001
**Smoking [*****N*** **(%)]**			
Yes	10,757 (37.2)	2,539 (35.3)	2,874 (39.5)	2,833 (39.4)	2,511 (34.7)	0.036
No	18,141 (62.8)	4,663 (64.7)	4,402 (60.5)	4,351 (60.6)	4,725 (65.3)	
**CVD [*****N*** **(%)]**			
Yes	3,531 (12.2)	671 (9.3)	819 (11.3)	920 (12.8)	1,121 (15.5)	< 0.001
No	25,367 (87.8)	6,531 (90.7)	6,457 (88.7)	6,264 (87.2)	6,115 (84.5)	
**Hypertension [*****N*** **(%)]**			
Yes	17,077 (59.1)	3,933 (54.6)	3,997 (54.9)	4,280 (59.6)	4,867 (67.3)	< 0.001
No	11,821 (40.9)	3,269 (45.4)	3,279 (45.1)	2,904 (40.4)	2,369 (32.7)	
**Education [*****N*** **(%)]**			
Lower	22,822 (79.0)	5,623 (78.1)	5,603 (77.0)	5,577 (77.6)	6,019 (83.2)	< 0.001
Higher	6,076 (21.0)	1,579 (21.9)	1,673 (23.0)	1,607 (22.4)	1,217 (16.8)	

N, number; BRI, body roundness index; MASLD, Metabolic dysfunction-associated steatotic liver disease; FBG, fasting blood glucose; Cr, Creatinine; SBP, Systolic blood pressure; UA, Uric acid; HDL-C, high-density lipoprotein cholesterol; CVD, Cardiovascular diseases.

Continuous variables are presented as mean ± standard deviation (normally distributed) or median with 25-75th percentile (not normally distributed).

### 3.2 All-cause mortality associated with BRI in MASLD

**Figure 2:**
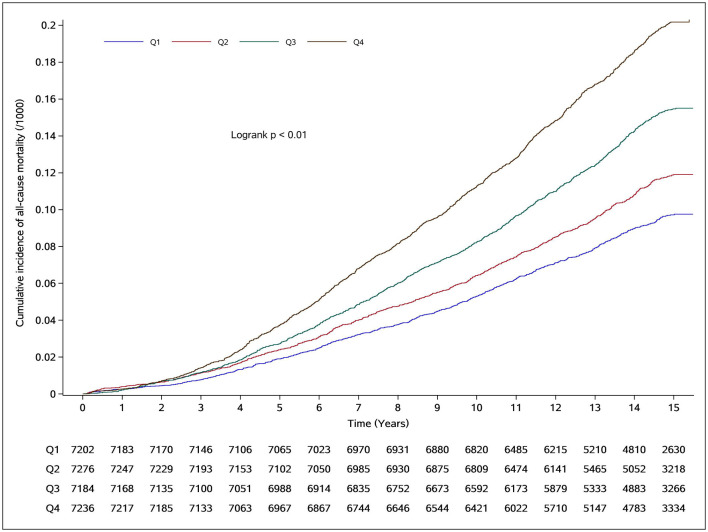
Cumulative incidence of all-cause mortality in different BRI groups.

During a median follow-up of 13.62 years (interquartile range: 12.85–15.16 years), a total of 3,895 deaths were documented. The study population included 22,998 men and 5,900 women. The all-cause mortality rates per 1,000 person-years increased progressively across BRI quartiles, from 6.58 in Q1 to 8.11 in Q2, 10.70 in Q3, and 14.33 in Q4. Correspondingly, the cumulative all-cause mortality rose from 9.04% in Q1 to 11.15%, 14.56%, and 19.17% in Q2–Q4, respectively. Kaplan–Meier analysis demonstrated significant differences in cumulative all-cause mortality across BRI quartiles (log-rank test, *P* < 0.001; Figure 2).

**Figure 3:**
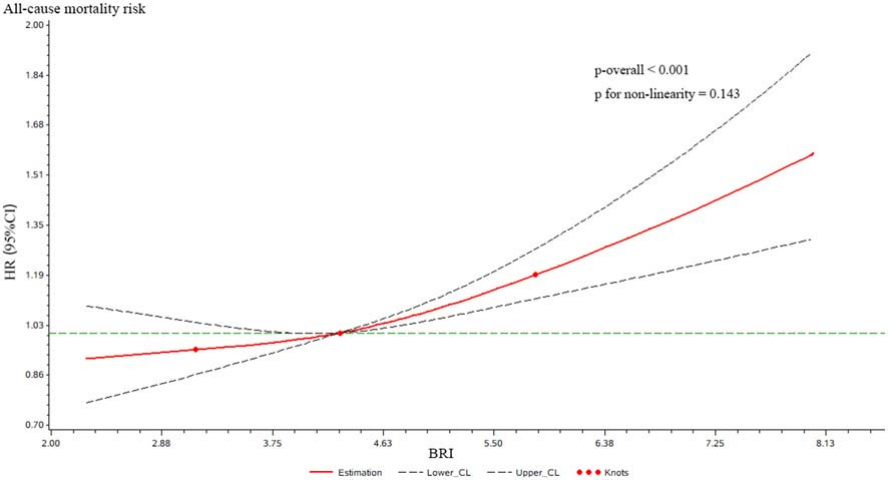
Restricted cubic spline curves for the impact of BRI on the occurrence of all-cause mortality in MASLD. Restricted cubic spline models were applied to evaluate the dose–response relationship between BRI and the risk of all-cause mortality. The solid red line represents the estimated hazard ratio (HR), and the dashed black lines indicate the 95% confidence interval (95% CI). Red dots denote the locations of spline knots. The green dashed line indicates the reference level (HR = 1).

Restricted cubic spline analysis showed a positive linear association between continuous BRI levels and all-cause mortality in the MASLD population (*P* for overall association < 0.001; *P* for nonlinearity = 0.143; Figure 3).

In Cox proportional hazards analyses, higher BRI quartiles were consistently associated with increased risk of all-cause mortality (Table 2). In the unadjusted model, the hazard ratios (HRs) for Q2, Q3, and Q4 compared with Q1 were 1.23 (95% CI: 1.11–1.37), 1.64 (95% CI: 1.48–1.80), and 2.20 (95% CI: 2.01–2.42), respectively. After adjustment for age and sex, the corresponding HRs were 1.02 (95% CI: 0.92–1.13), 1.12 (95% CI: 1.02–1.24), and 1.24 (95% CI: 1.12–1.36). Further multivariable adjustment attenuated the associations but they remained statistically significant for higher BRI categories, with HRs of 1.05 (95% CI: 0.94–1.18), 1.14 (95% CI: 1.02–1.28), and 1.21 (95% CI: 1.06–1.37) for Q2–Q4, respectively (*P* for trend < 0.001). When BRI was analyzed as a continuous variable, each standard deviation increase in BRI was associated with a 13% higher risk of all-cause mortality (adjusted HR: 1.13; 95% CI: 1.07–1.19; *P* < 0.001).

**Table 2:** COX proportional hazards model analysis of the effect of BRI level on all-cause mortality in MASLD.

**Quartile**	**Event/Total population**	**Incidence density/103 person-years**	Model 1		Model 2		Model 3	
			**HR (95% CI)**	***P*** **value**	**HR (95% CI)**	***P*** **value**	**HR (95% CI)**	***P*** **value**
Q1	651/7,202	6.58	Ref		Ref		Ref	
Q2	811/7,276	8.11	1.23 (1.11–1.37)	< 0.001	1.02 (0.92–1.13)	0.681	1.05 (0.94–1.18)	0.389
Q3	1,046/7,184	10.70	1.64 (1.48–1.80)	< 0.001	1.12 (1.02–1.24)	0.024	1.14 (1.02–1.28)	0.025
Q4	1,387/7,236	14.33	2.20 (2.01–2.42)	< 0.001	1.24 (1.12–1.36)	< 0.001	1.21 (1.06–1.37)	0.005
*P* for trend			< 0.001		< 0.001		< 0.001	

Model 1: Single factor model.

Model 2: Corrected for age and gender based on Model 1.

Model 3: Corrected for Hs-CRP, FBG, HDL-C, CR, SBP, UA, Neutrophil, hip circumference, smoking status, cardiovascular disease history, hypertension history, and educational attainment based on Model 2.

### 3.3 Stratified analysis

Stratified analyses were performed to examine the association between BRI and all-cause mortality across different characteristics of the MASLD population. Cox proportional hazards models were constructed with BRI quartiles (Q1 as the reference) as the exposure and all-cause mortality as the outcome, adjusting for the same covariates as in Model 3. Stratification was conducted according to age, sex, hypertension status, and education level. Among participants younger than 65 years, higher BRI levels were associated with increased all-cause mortality risk. Compared with Q1, the adjusted hazard ratios (HRs) for Q2, Q3, and Q4 were 1.10 (95% CI: 0.96–1.27), 1.19 (95% CI: 1.03–1.38), and 1.38 (95% CI: 1.17–1.63), respectively. In other subgroups, including individuals aged ≥65 years, those with education beyond high school, and non-hypertensive participants, the associations between BRI quartiles and all-cause mortality were not statistically significant. Interaction terms between BRI and subgroup variables were tested in the Cox proportional hazards models. A significant multiplicative interaction was observed for sex, whereas no significant interactions were found for age, educational attainment, or hypertension (Table 3).

**Table 3:** Stratified analysis of BRI and risk of all-cause mortality.

**Subgroups**	**BRI**	**Event/Total population**	**Incidence density/ 10^3^person-years**	**HR (95%CI)**	***P* for trend**	***P* for interaction**
**Age, years**			
< 65	Q1	426/6,690	4.58	Ref	< 0.001	0.250
	Q2	461/6,490	5.08	1.10 (0.96–1.27)		
	Q3	505/6,048	5.97	1.19 (1.03–1.38)		
	Q4	584/5,558	7.55	1.38 (1.17–1.63)		
≥65	Q1	225/512	38.47	Ref	0.685	
	Q2	350/786	38.04	0.96 (0.80–1.16)		
	Q3	541/1,136	40.93	1.07 (0.90–1.28)		
	Q4	803/1,678	41.26	1.02 (0.84–1.24)		
**Gender**			
Male	Q1	602/5,993	7.34	Ref	0.006	0.017
	Q2	744/5,964	9.15	1.06 (0.94–1.19)		
	Q3	946/5,776	12.17	1.15 (1.02–1.30)		
	Q4	1,156/5,265	16.74	1.17 (1.02–1.34)		
Female	Q1	49/1,209	2.89	Ref	0.009	
	Q2	67/1,312	3.59	0.96 (0.64–1.45)		
	Q3	100/1,408	4.99	1.11 (0.74–1.65)		
	Q4	231/1,971	8.33	1.57 (1.04–2.35)		
**Education**			
Below high school	Q1	582/5,623	7.50	Ref	0.001	0.058
	Q2	718/5,603	9.33	1.07 (0.95–1.21)		
	Q3	903/5,577	11.89	1.16 (1.03–1.32)		
	Q4	1,232/6,019	15.33	1.22 (1.06–1.39)		
Higher	Q1	69/1,579	3.23	Ref	0.138	
	Q2	93/1,673	4.04	0.86 (0.59–1.24)		
	Q3	143/1,607	6.56	0.97 (0.68–1.40)		
	Q4	155/1,217	9.45	1.15 (0.77–1.73)		
**Hypertension**			
Have Hypertension	Q1	492/3,933	9.16	Ref	< 0.001	0.549
	Q2	594/3,997	10.95	1.00 (0.87–1.14)		
	Q3	800/4,280	13.90	1.12 (0.98–1.28)		
	Q4	1,145/4,867	17.89	1.22 (1.05–1.41)		
NO Hypertension	Q1	159/3,269	3.51	Ref	0.216	
	Q2	217/3,279	4.75	1.25 (1.00–1.57)		
	Q3	246/2,904	6.12	1.19 (0.94–1.50)		
	Q4	242/2,369	7.38	1.21 (0.93–1.58)		

BRI, body roundness index; HR, hazard ratio; CI, confidence interval.

### 3.4 Sensitivity analyses

Sensitivity analyses assessed the robustness of primary findings. Analysis 1 excluded 179 participants who died within 2 years of enrollment, with Cox models adjusted for Model 3 covariates. Compared to Q1, adjusted HRs (95% CIs) for Q2–Q4 were 1.03 (0.92–1.15), 1.11 (0.99–1.24), and 1.21 (1.07–1.37) (*P* for trend < 0.001). Analysis 2 included 1,756 cancer patients, yielding HRs of 0.99 (0.90–1.09), 1.07 (0.97–1.18), and 1.19 (1.07–1.33) (*P* for trend < 0.001) for Q2–Q4 versus Q1 after full adjustment (Table 4).

**Table 4:** Sensitivity analysis.

**Quartile**	Sensitivity analysis 1				Sensitivity analysis 2			
	**Event/Total population**	**Incidence density/ 10** ^3^ **person–years**	**HR (95% CI)**	***P*** **value**	**Event/Total population**	**Incidence density/ 10** ^3^ **person-years**	**HR (95% CI)**	***P*** **value**
Q1	619/7,170	6.26	Ref		852/7,631	8.20	Ref	
Q2	764/7,229	7.65	1.03 (0.92–1.15)	0.655	981/7,669	9.37	0.99 (0.90–1.09)	0.859
Q3	997/7,135	10.20	1.11 (0.99–1.24)	0.072	1,261/7,637	12.24	1.07 (0.96–1.17)	0.239
Q4	1,336/7,185	13.81	1.21 (1.07–1.37)	0.003	1,658/7,680	16.31	1.19 (1.06–1.31)	0.004
*P* for trend	< 0.001				< 0.001			

Sensitivity analysis 1: excluded 179 participants with less than 2 years of death, adjusted for confounders as in model 3.

sensitivity analysis 2: reserving 1,756 participants reporting a history of cancer, adjusted for confounders as in model 3.

## 4 Discussion

In this prospective cohort study of 28,898 participants from the Kailuan Study followed for over 10 years, we found that BRI was positively associated with all-cause mortality in the MASLD population. Each standard deviation increase in BRI was associated with a 13% increased risk of all-cause mortality among MASLD patients. To our knowledge, this is the first study to evaluate BRI association with all-cause mortality in a Chinese MASLD population. These findings highlight the importance of managing visceral fat health in patients with MASLD.

Compared with traditional anthropometric indices such as body mass index (BMI), which primarily reflects overall adiposity, Body Roundness Index (BRI) incorporates waist circumference and height to better characterize body shape and central fat distribution (17). BMI does not distinguish fat mass from lean mass and fails to capture visceral fat accumulation, a key driver of metabolic dysfunction. In contrast, the body roundness index (BRI) has been proposed as a practical surrogate marker of visceral adiposity, and subsequent studies have demonstrated that BRI exhibits stronger associations with cardiometabolic multimorbidity than BMI (25).

Within the MASLD population, two-thirds of individuals exhibit three or more cardiometabolic risk factors, with obesity being one of the most prevalent (26). Visceral adiposity, in particular, is considered an established risk factor associated with cardiovascular events and all-cause mortality. Although waist circumference is commonly used to assess central obesity, it does not account for body frame or height-related differences, whereas BRI integrates these geometric features and may provide a more refined assessment of body roundness and visceral fat burden. Currently, there is growing recognition that visceral fat confers greater health risks than subcutaneous fat due to its heightened disease burden (27, 28). While the rationale for using BRI to estimate visceral fat distribution may be sound, evidence linking BRI to disease or mortality remains limited. Wu et al. (29) demonstrated dose-dependent increases in cardiovascular event risk with higher BRI among 59,278 participants without malignancy or cardiovascular disease, particularly in younger individuals. Zhang et al. (22) reported rising BRI trends over nearly two decades and a U-shaped between BRI with all-cause mortality association in 32,995 US adults from NHANES (1999–2018). Yi et al. (23) reported that higher BRI values were associated with an increased risk of all-cause and cardiovascular mortality among individuals with MASLD. To complement previous research, we specifically focused on a Chinese population with MASLD and observed a positive association between BRI and all-cause mortality in this population. This association remained robust in sensitivity analyses after excluding participants who died within the first 2 years of follow-up and retaining participants with cancer at baseline. Therefore, our findings may help inform clinical decision-making.

In this large cohort, we observed a stronger association between BRI and all-cause mortality among adults under 65 years, underscoring the need for enhanced visceral fat management in this demographic. Conversely, no significant association was found between BRI and all-cause mortality in individuals aged≥65 years. This differential association appears biologically plausible given BRI's role as a potential nutritional status surrogate (30), where extremely low BRI values correlate with malnutrition, fatigue, reduced exercise tolerance, and muscle wasting. In older populations, moderate obesity may reflect better nutritional status and greater metabolic reserves, which could confer a survival advantage under conditions of chronic illness or acute physiological stress, thereby contributing to a lower risk of all-cause mortality—an observation commonly referred to as the “obesity paradox.” (31, 32). Epidemiologically, elevated BRI significantly associates with increased risks of cardiovascular/metabolic disorders and cancer (21, 33–36), potentially representing a major contributor to all-cause mortality. Clinically, visceral fat accumulation links to aggravated insulin resistance and elevated cardiometabolic risk, even in participants within normal body weight ranges (37, 38), collectively contributing to excess mortality. Future research with more detailed phenotypic characterization and longitudinal assessments is warranted to further elucidate the mechanisms underlying the observed association between fat distribution and all-cause mortality in older adults.

Sex-stratified analyses showed that BRI was associated with all-cause mortality in both men and women, with a stronger association observed among women. This finding may be related to hormonal changes around menopause, as the mean age of female participants approximated the menopausal transition in Chinese women. Declining estrogen levels are known to promote visceral fat accumulation and worsen cardiometabolic risk, potentially amplifying the adverse effects of elevated BRI on mortality (39). In contrast, no significant associations were observed among individuals with higher educational attainment or without hypertension. Higher education may be linked to healthier behaviors and better disease management, while non-hypertensive MASLD patients may exhibit less severe metabolic disturbances, resulting in lower mortality risk (26, 40).

This study has several strengths, including its prospective cohort design, large sample size, long-term follow-up, reliable physiological and biochemical measurements, and comprehensive collection of lifestyle data. We demonstrated a positive association between BRI levels and all-cause mortality in the MASLD population through a long-term population-based prospective cohort study. Despite these valuable findings, certain limitations exist. First, as an observational study, causality cannot be definitively established. Second, while ultrasonography for MASLD assessment is considered safe, accurate, and practical for large-scale epidemiological studies, it may be less accurate than liver biopsy. Third, due to funding constraints and study design, we lacked data on aspartate aminotransferase and liver biopsy, limiting our ability to accurately or indirectly determine liver fibrosis staging. Fourth, due to limitations in data collection, we were unable to comprehensively and accurately ascertain specific causes of death. Finally, as the Kailuan cohort consists predominantly of male industrial workers, the generalizability of our findings may be limited to Chinese populations and may not fully extend to women, non-urban populations, or individuals from other ethnic or geographic backgrounds, warranting further validation in diverse populations.

## 5 Conclusion

In this large cohort study, our findings demonstrate a significant positive association between BRI and all-cause mortality in the MASLD population. As a non-invasive and readily accessible screening tool, BRI may provide valuable insights for optimizing risk management strategies in future MASLD clinical practice.

## Data Availability

The datasets presented in this article are not readily available because According to the requirements of the institution, the dataset of this study is not made public. Requests to access the datasets should be directed to Xiangming Ma brighter_ma@163.com.
